# The Effect of Sound in the Dental Office: Practices and Recommendations for Quality Assurance—A Narrative Review

**DOI:** 10.3390/dj10120228

**Published:** 2022-12-05

**Authors:** Maria Antoniadou, Panagiota Tziovara, Christina Antoniadou

**Affiliations:** 1 Dental School, National and Kapodistrian University of Athens, 11527 Athens, Greece; 2 Royal Academy of Music, University of London, London WC1E 7HU, UK

**Keywords:** noise, dental office, sound effect, healthcare, music in healthcare, healing music, occupational noise, dentistry

## Abstract

Sound is inextricably linked to the human senses and is therefore directly related to the general health of the individual. The aim of the present study is to collect data on the effect of two dimensions of sound, music, and noise from an emotional and functional point of view in the dental office and to perform a thorough review of the relevant literature. We collected articles from the databases PubMed and Google Scholar through keywords that were related to noise and music in healthcare. Important information was also extracted from articles on the web and official websites. Screening of the relevant literature was performed according to accuracy and reliability of the methodology tested. A total of 261 articles were associated to sound and music in healthcare. Ninety-six of them were the most well documented and were thus included in our article. Most of the articles associate noise with negative emotions and a negative impact on performance, while music is associated with positive emotions ranging from emotional state to therapeutic approaches. Few results were found regarding ways to reduce noise in a health facility. If there is a difficulty to find effective methods of reducing the daily noise-inducing sounds in the dental office, we must focus on ways to incorporate music into it as a means of relaxation and therapy.

## 1. Introduction

Dental staff and patients in the dental office are exposed to a barrage of sounds [1]. Sound can have either a negative impact, perceived as noise [2], or a positive one, for example while listening to pleasant music [3]. The dental office is a health working environment with external sounds that can be limited but not completely controlled or extinguished and internal ones that derive from physical movements, communications and dental equipment activities in the waiting and operational rooms. Anxious and phobic patients are common phenomena in dental healthcare units [4], and they are especially sensitive to sound and touch [5]. Dental anxiety has been already defined as a state of worry, nervousness, or unease for a dental procedure with an uncertain outcome [6] that can worsen with loud noise [7]. Anxious patients can become uncooperative and potentially more difficult to manage, as they tend to avoid dental visits. Consequently, they suffer from dental diseases such as caries, represented with pain [8]. Age can play a certain role in the estimation of noise and the consequent sentiments it brings. Especially for children, feared aspects of dental treatment are often sensory, such as the sound of a dental drill [7,9]. Furthermore, music listening offers an effective, nonpharmacologic alternative to reducing preprocedural dental anxiety in patients with intellectual and developmental disabilities (IDD) [10]. In their systematic review, Moola et al. [11] concluded that there was enough evidence to suggest that adult patients may also benefit from a procedural music-listening program but there was inconclusive evidence on the effectiveness of music in reducing dental anxiety in children. Pathological issues with sound are also described in the relevant literature, with misophonia being one of the most searched disorders [12]. In misophonia, certain sounds trigger emotional or physiological responses that could be perceived as unreasonable for others under the given circumstances. In such cases, a sound such as a dental drill may “drive dental patients crazy”. They can range from anger and annoyance to panic and the need to leave the office [5].

Inversely, a therapeutic approach to sound in the dental office is a topic of increasing interest in the literature. Music can be used as a self-management technique to reduce or control distress [13]. Music therapy is an effective, non-invasive, and cost-effective intervention, which decreases anxiety and therefore optimizes the outcome of a medical intervention [6]. Music is seen to have wellbeing benefits but also many advantages in health, across a spectrum of practices: from community settings to its use in waiting rooms and surgical settings as background music, both to directly influence mood and arousal levels and to distract from unpleasant thoughts and feelings [5,7]. Through music, great changes can be observed in mood improvement, pain and anxiety masking, cardiovascular fitness enhancement, mindfulness or/and greater social integration [7]. According to the literature and to the best of our knowledge, little attention has been paid to sound’s impact in the therapeutic procedure of dental patients and the relaxation state or health of both patients and dental staff. The aim of this article is then to perform a narrative review on the effects of two dimensions of sound, music and noise, to both patients and dental professionals, from an emotional and functional point of view. We further proceed to suggested technical actions for soundproof design of modern dental offices as well as personal actions of sound control and music use for the wellbeing of dental staff and patients.

## 2. Materials and Methods

**Figure 1:**
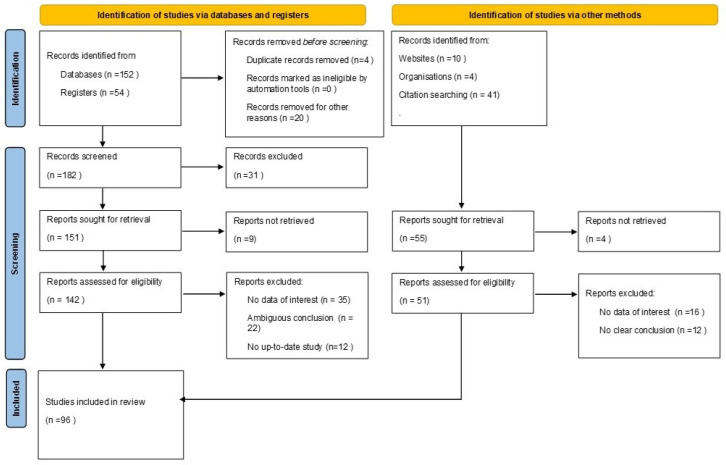
Schematic view of the methodology process.

For the purpose of this narrative review, we performed research in databases and google searching with keywords such as “noise”, “dental office”, “sound effect”, “healthcare”, “music in healthcare”, “healing music”, “occupational noise” and “dentistry”. From this search, we found 261 articles discussing the aspects of sound and music in healthcare settings. From these articles, 165 were excluded because of insufficient, irrelevant or ambiguous conclusions and data. From the remaining articles, 45 were performed in hospitals, and 41 were performed in dental settings. From the screened articles, we used the ones that had scientific references and were well-documented, which had a final total of 96. A total of 4 of the 96 references led to official foundations and organizations and 3 to official websites. All the above are presented in the following Prisma flow chart (Figure 1) [14].

## 3. Sound Types and Levels in the Dental Office

The sounds received in a dental office can be mainly divided into two main groups. The first group includes sounds from non-dental external sources, such as traffic noise, roadworks, etc., and sounds inside the dental clinic, for example phone ringing, conversations between the staff and patients, air conditioner, computer printers, music, and television [14,15]. The exposure of the patient and staff to these sounds can be limited but not completely controlled. The second group includes sounds from dental sources, i.e., sounds produced by various machines that operate continuously or intermittently. For example, high-speed drills [16,17,18] and cutting machines in general [19], ultrasonic scaling handpiece [19,20], power suction equipment [19,20,21], amalgamators, autoclave laser equipment and other instruments and trolleys [1,22]. The noise levels in the dental operating room have been linked previously to those encountered on a motorway [23].

According to the National Research and Safety Institute, for an 8 h workday, hearing is in danger from 80 dB [24]. The noise levels in the dental clinics are below the limit of risk of hearing loss [3,21,22,23]. This is attributed to the development of modern dental equipment and machines that considerably reduce the degree of noise produced. To decrease the prevalence of hearing loss among dental professionals, ISO standard 7785:199716 suggests that the noise levels (namely, sound pressure levels) generated by the high-speed handpieces should be below 65 dBA and should never exceed 80 dBA. According to this ISO, the noise levels produced by new dental equipment are generally below 85 dBA [22,23].

Thus far, there have been several studies in the literature that have examined the dBA level produced by sounds in the dental office. In those studies, noise levels associated with clinical handpieces ranged from 76 to 105 A-weighted decibels (dBA), and suction ranged from 74 to 80 dBA in a 1979, United States Army study [25], such as levels of 70–82 dBA for clinical handpieces and 82–90 dBA for cleaners and scalers reported in a separate study [26]. Reports in the contemporary literature suggest that noise levels may have declined substantially over the intervening 30+ years; mean levels of 70–76 dBA for clinical handpieces and suction were reported in 1998 [23], 66–76 dBA in 2006 [27], 64–97 dBA in 2011 [28], 64.2 +/− 2.4 dB in 2013 [29] and 75–84 dBA in 2014 [30] or 51.7–67.37 dBA [31] and 60–65 dBA in 2017 [19]. Most of these measurements were brief measurements made near operating dental instruments, rather than measures of personal exposure [32] or in a big clinic with more than one dental unit. In another research set up, measures have been made in different spots within a clinic suggesting that mean sound levels in the working clinics ranged from 63.0 to 81.5 dBA, being within the suggested limit. In the same study, the combination suction and either low- or high-speed handpiece in the postgraduate clinic was significantly noisier than the undergraduate clinic at several times, suggesting that more intensive dental work might generate more noise [33]. Furthermore, in a recent study, an overall noise of 73.83 ± 4.39 dB was found to be generated within a dental clinical setting, suggesting that in clinics with more than one dental unit, especially when dentists perform different operational activities, sounds remain high and close to average limits of risk [34]. More specifically, the highest sound level of 79.44 ± 2.10 dBA was observed during restorative treatment followed by 74.14 ± 3.08, 73.22 ± 1.93, 71.39 ± 3.37 dBA for endodontic, periodontal, and prosthodontic treatments, respectively. A statistically significant difference was observed in the noise levels produced from all these different specialty treatments [34].

The extensive reports made by organizations such as the World Health Organization, the Control of Noise at Work Regulations in the United Kingdom and the US Occupational Safety and Health Administration (OSHA) are also interesting. National and European community directives and United Nations guidelines apply for workplaces including operation theatres. The recommended threshold for work, which is characterized by a significant part of mental activity such as decisions under time pressure, decisions with severe consequences, is 55 dBA [1]. Noise levels frequently exceed recommended noise levels by the World Health Organization in hospitals, especially in the operating room [35], as it recommends sound levels up to 35 dBA [36]. Although average sound levels do not exceed the thresholds recommended by law and international standards, momentary peaks are higher than the allowable level. Therefore, it is essential to introduce means of prevention and measures of safety against the daily dental exposure to noise [24].

## 4. Effect of Noise in the Dental Office

Noise is defined as an unpleasant and unwanted sound [5]. For dental patients, the most studied effect of noise is anxiety. Fear or anxiety due to noise produced in the dental clinic is rated third among the reasons to avoid dental visits [18]. Patients often associate the dental office as an unfriendly, offensive, and anxiety-provoking environment, distinguished by loud noises [17]. All patients’ age groups can be annoyed or stressed from noise derived from dental equipment, with children being most affected. Among children with adverse responses to sensory stimuli, noise was the most adverse stimulus, followed by touch, smell and backward tilting of the examination chair. [37]. While one study found that noise from a high-speed drill and the Erbium laser did not cause any irritable behavior among children, Yu et al. [20] showed that noise from the ultrasonic scaling handpiece was perceived as an aversive auditory stimulus by young patients and induced unpleasant feelings. In another study, 38% of the patients in the age group 6–11 years reported that the sound of the drill makes them uncomfortable [18]. Sound and sensation of the drill were rated as the most fear-eliciting stimuli in children [17,18] and cause of dental anxiety. In older children, dental anxiety is found to be higher, with 76% of 12-year-olds and 64% of 15-year-olds reporting either moderate or severe dental anxiety when visiting the dentist [7].

Similarly, noise in the dental office affects clinicians and auxiliary dental staff as well. Occupational noise is the most frequently studied type of noise exposure in the dentistry field [38]. Noise can affect the hearing capability of dental professionals. Auditory disorders, tinnitus and hearing damage are common harmful effects of prolonged exposure to noise in dental settings [22]. In modern studies, a large discrepancy in the prevalence of hearing loss for professionals of the dental field was found, while elsewhere, no hearing loss was noticed [19]. All researchers agree, however, that noise levels in the dental office are high enough to cause other non-auditory negative effects, such as annoyance, anxiety, irritation, conversation interference and concentration difficultly [19], as well as fatigue and tension headaches [38,39]. Dentists with a service length of more than 10 years and daily working hours of more than eight were found to have the highest risk to their hearing state. In addition, the worse the hearing state was, the worse the health state was found for them [40]. Elsewhere, hearing loss was significantly related to work tenure longer than 15 years and age older than 40 years in a dental population [41].

External noise pollution is regarded further as a general stressor for both patients and staff. It increases mental stress, the development of cerebral cardiovascular disease, and the risk of hearing loss [35,42]. Volume level and the frequency of noise (sound quality) have negative effects on concentration as well. Furthermore, external noise can bring adverse effects that range from poor concentration to mental and physical stress both subjectively and objectively in an already stressful environment, plagued with high burnout levels [1,35,43]. Higher volumes of noise correlate directly with higher levels of surgical errors, putting patients at risk. The more complex the operation procedures are, the more severe the negative effects of external noise become [44]. 

Noise pollution in an operational room can also be caused by employee-related behaviors and surgical equipment. Communication was the factor believed to be most adversely affected by noise in the operating room [14,35]. The noise caused disruption and ‘‘masking’’, which resulted in impaired speech discrimination and speech intelligibility. Consequently, the staff raised their voices to be well understood, which amplified the noise level [2].

The effect of noise on performance depends not only on its level and the stress tolerance of the individual, but also on the complexity of the task and the type of noise. Two features of the type of noise are important, namely, whether it is predictable and whether it is controllable. Predictable noises are continuous or periodic, and unpredictable ones are discontinuous or episodic; controllable noises can be terminated at will, whereas uncontrollable ones cannot. Even high-level continuous noises (90–120 dBA) have no detrimental effects on the performance of simple motor or mental tasks. However, noises of lesser amplitude, especially when they are unpredictable, uncontrollable, or both, can interfere substantially with the performance of complex tasks [1]. In general, unexpected suddenness of noise occurrence shows significant influence on dental works. In comparison, frequency of noise occurrence has a less but still significant influence on dental work, particularly on the quality of work and necessary conversation among staff. However, noise level shows a weak influence on dental staff, and noise from their own equipment shows a secondary influence [19].

## 5. Positive Aspects of Sound in the Dental Office

The healing effect of sound is traced back thousands of years. From the Aborigines who used the powerful sound vibrations of the “yidaki” (wind instrument) that would help listeners to enter a deep state of relaxation, to the Tibetan monks using the Himalayan bowls whose vibrations were described as universal manifestation, and from American Indian healers who would fast in order to dream of a healing song for their patient [45], to the ancient Egyptians who developed a method that called “toning” (a method aiming into manipulating the sound of vowels in order to create therapeutic sounds) [46], and to the ancient Greeks who used music to improve wellbeing [3], sound has always been an extraordinary tool of healing, both physical and emotional [47,48]. There has been a rising interest in the therapeutic power of music in healthcare in the last two decades. Music has been used in different medical fields to meet physiological, psychological, and spiritual needs of patients [11]. Specifically, the anxiolytic effects of music have been studied in a variety of medical patients, including surgical, cardiac, oncology, and urology patients as well as dental patients [49]. 

Music has many benefits as far as concerning relaxation matters, and it can have a positive influence on the patient by making concentration easier and by easing anxiety [50]. Many scientists have investigated the therapeutic effect of music on patients before, during and after surgical procedures of different kinds. It generally proves to be useful: (a) for reducing anxiety and pain levels, (b) during the recovery period [3,51,52], and (c) for encouraging people to commit to routine and necessary preventive care [53]. In the field of dentistry, a systematic review concluded that music intervention was effective in reducing anxiety and pain in children undergoing dental procedures and in adults undergoing medical procedures [13,50,54,55]. In another study, patients were asked what they would recommend as a useful way of reducing discomfort during dental procedures. The most frequent response was the use of music [56]. A 10 min music intervention is a sufficient period to allow music to exert an anxiolytic effect. This is an important finding, as waiting periods in dental or other medical offices are rather short in time [57] or should be. Moreover, it was found that music listening is equally effective in decreasing anxiety levels or even to a greater extent than the administration of benzodiazepines [57]. Despite the broad range of settings and patients in which music intervention is tested, it has not yet been broadly adopted in clinical practice [3].

Music also has a positive effect on the performance of the healthcare staff. It brings higher satisfaction within the working environment, which is associated with a lower chance of a burnout [35]. It is reported that the only form of noise that can be beneficial in the operating room is music, which may raise the concentration when experienced operators perform a monotonous task [42]. Moreover, it is believed that general conversation and music should be acceptable, as this increases work enjoyment in an already stressful environment and prohibiting it entirely would not be feasible [35]. Although the “sterile cockpit concept” is often mentioned (a room isolated from external noises), a total sound-sterile work environment in the operational room seems to be neither practically possible nor desirable [35]. In addition, stress-reductive effects of music in healthcare professionals have been described [58] making music an effective way of happiness, excellence and productivity in the workplace that positively affects the surgical performance and the postoperative complications of a difficult dental procedure such as implantology (Sound Healing Research Foundation) [59]. On the other hand, in a clinical trial, 20% of the responders viewed music as a distracting factor when played during a long, complicated, or emergency procedure. Music influenced communications positively between staff as reflected by 63% of the responders, and 77% reported that music made them calmer and more efficient. Those who refused to listen to music during surgery indicated that it might interfere in extended and complicated procedures as well as in emergency procedures [51].

In terms of the type of music proposed, a study concluded that the anxiolytic and pain-reducing effects are not restricted to one specific type of music [3]. For example, classical music was preferred by 58% of the responders [51]. A meta-analysis concluded that there is a small but statistically significant beneficial effect of listening to Mozart on task performance [60]. However, this effect can also be observed with other types of music [52]. Thus, the delivery of music that is appropriate in dental settings is another important issue of research. The brain of each individual patient has picked up musical building blocks from the local sonic environment in infancy and developed preferences based on this experience. To the extent possible, music needs to be tuned to resonate with patients’ particular and deep-rooted musical instinct. The evidence for this is overwhelming patient preferences, and prior musical experiences are vital determinants of the ultimate success of any intervention. Ideally, music should be relevant to its listeners in terms of culture, genre, mood, and era of origin [61]. Because music is an inherently evocative medium, dental professionals also need to be cautious not to evoke too much feeling or irritation instead of relaxation.

The volume of music is also important in healthcare units. Some people need high volume music to remain calm, whereas others feel overwhelmed by the very same sensory experiences [37]. Another issue is who is delivering the music program. Ullmann et al., in 2008, mentioned that speed and accuracy of a task performed is greater when the surgeon selects the music [51], and this is also mentioned elsewhere [52]. The same effect is found for patients as well. In a relevant study, it was reported that music is most effective when the musical program is selected by the patient [56]. There is a distinction between music interventions administered by medical or healthcare professionals (passive music listening) and those implemented by trained music therapists (active music therapy) or those performing sound baths for mindfulness and relaxation. Active music therapy is the planned and creative use of music by a music therapist to attain and maintain health and wellbeing. People of any age or ability may benefit from a music therapy program regardless of musical skill or background [11,61].

Specifically in the dental field, it was concluded that music alone did not produce any quantifiable distraction affecting pain, anxiety, or patient behavior in dental patients [62]. However, patients enjoyed listening to the music during their visits. Another systematic review mentions that music relaxation administered prior to dental treatment yielded no dental-anxiety-reducing effect compared to the control group that was resting in silence [63]. Furthermore, several dental studies have attempted to evaluate the use of audio and video distraction as an adjunct to dental treatments [62]. Elsewhere, it was mentioned that adult dental patients reported reduced pain and reduced anxiety with video distraction and audiotaped relaxation instructions, but not with music, which at best, results in a placebo effect [56]. An innovative approach to these methods that has many benefits to the management of pain and anxiety is virtual reality [64], more extensively used currently [65], as it results in an audiovisual distraction [18]. As it is mentioned thus far, the distraction with music and audio–visual interventions may be more effective for patients with mild forms of anxiety compared to patients with severe dental anxiety. This is because distraction therapies operate on the principle of masking of fear-stimuli prior to or during dental treatment and do not facilitate learning in patients with dental anxiety [63]. This is extremely important in cases of needle-related procedures that cause pain and distress (especially common during childhood). During these moments, it is recommended for the dental staff to control the level of sound of the background music either through the headphones or other audio–visual equipment to cause distraction, relaxation through deeper breathing and creation of pleasant new memories [66]. It is also recommended that pre-recorded music could be offered through headphones during stressful procedures to adult patients to reduce their dental anxiety too [11].

Finally, guidelines have been issued that describe the therapeutic use of music in healthcare [61]. In the protocol of the Music Therapy Unit, of the Royal Children’s Hospital in Melbourne (2004) [67], it is mentioned that: (a) radio is a source of uncontrollable stimulation. Radio use should be limited to use with headphones; (b) background recorded music should be controlled by a member of staff (for example waiting areas), and the situation should be regularly assessed (i.e., every few hours) and altered where not appropriate; (c) patients should be encouraged to bring and use music they consider to be a supportive strategy.

Selected literature on music effects in dental settings are shown in Table 1. We selected the most up-to-date, well-organized articles with high scientific value. Regarding operators and staff, music shows a positive influence on behavior and emotional state and is preferable while working. Working is more enjoyable, but surgical performance is not shown to be affected either positively or negatively. Concerning patients, listening to music is helpful prior to dental treatment but also during the procedure, without affecting the levels of pain.

## 6. Mechanism of Healing Effect of Sound and Music in the Dental Office

It is reported that music engages sensory processes, attention, memory-related processes, perception–action mediation (“mirror neuron system” activity), multisensory integration, activity changes in core areas of emotional processing, processing of musical syntax and musical meaning, and social cognition [70]. Music has been shown to stimulate the brain primary engines of human capacity. Musical engagement exercises, attentional networks, and executive function evoke emotional response, stimulate the central nervous system, and appear to activate the human mirror-neuron system, supporting the coupling between perceptual events (visual or auditory) and motor actions (leg, arm/hand, or vocal/articulatory actions). It has been used successfully to induce cognitive repair in patients with stroke, Parkinson’s disease, cerebral palsy, or traumatic brain injury. It is reported that music has the potential to fix the brain, by providing an alternative entry point into a broken brain system to remediate impaired neural processes or neural connections [61]. It is likely that the engagement of these processes by music can have beneficial effects on the psychological and physiological health of individuals, although the mechanisms underlying such effects are currently not well understood [71,72]. Experiment, measures of physiological response, and imaging together show that creating or listening to music engages regions throughout the brain, bilaterally, and in the cortex, neo-cortex, paleo-, and neocerebellum [73,74], distinguishing eight perceptual dimensions of music: pitch, rhythm, tempo, timbre, meter, contour, loudness, and spatial location, each of which has been tested independently by experiments. Each of these energetic components of music has been shown to activate distinct brain structures and neural circuitry. The current diagram of musical perception is of a sequence, wherein the base components of pitch, rhythm, and loudness are processed individually and separately within the brain [74,75] and then synthesized to create the understanding of an entire phrase [76]. Then, music healing effects run from its intervention in the brain’s regions that control emotions through the production of certain hormones [77].

Today, the research in the field of psychoacoustics, the scientific study of the perception of sound, has led scientists to analyze the brain waves that are triggered by different sounds. They found that specific sounds of different frequencies can touch off brain waves, which as electric pulses, can vibrate and work into the interaction of masses of neurons. All this activity can boost good hormones such as serotonin and can lower cortisol, which is associated with stress [48].

Dr. Margaret Patterson and Dr. Ifor Capel worked on a series of experiments on how Alpha brainwaves can boost serotonin. As Dr. Capel stated: “As far as we can tell, each brain center generates impulses at a specific frequency based on the predominant neurotransmitter it secretes. In other words, the brain’s internal communication system—its language, is based on frequency. Presumably, when we send in waves of electrical energy at, say 10 Hz, certain cells in the lower brain stem will respond because they normally fire within that frequency range” [45]. In another research by the British Academy of Sound Therapy, results have shown that sound is involved in the different domains of “physical relaxation, imagery, ineffability, transcendence of time and space, positive mood, insightfulness and disembodiment and unity both in live and recorded studies” and causes deep relaxation [78]. Especially, during sound baths, people come to the so-called “altered state of consciousness” (ACS); a state as if daydreaming or just before falling asleep. In this ACS state, the theta brain waves are synchronized with the healing sounds and provide the person with a state of deep relaxation that lasts awhile [79] and can alter their behavior during stressful events.

## 7. Options of Sound Control Design in the Dental Office

There are ways in which we can practically reduce the sounds coming from all the sources mentioned above or, in cases where this is not possible, we can get the person to pay less attention to them [80]. There are practical suggestions in the literature characterized as positive distractions that can be applied relatively easily, ranging from the design of a dental clinic to the daily management of the equipment and machines: (1) art and environmental aesthetics, (2) spatial arrangement and atrium, (3) considerations of socialization patterns, (4) play and interactive technologies, (5) sound and lighting interventions, and (6) access to nature. Relaxation music can combine all these interventions, especially in dental settings where fear is the main emotion. The research indicated that such positive distractions in healthcare environments provide a series of health benefits for patients, including improved behavioral and emotional wellbeing, reduced stress and anxiety, enhanced healthcare experience and satisfaction, facilitated therapeutic procedures, and postoperative outcomes that enhance quality assurance in the setting [81]. However, significant research gaps are emerging between positive distractions and play/relaxation in garden spaces within the waiting areas, suggesting that spatial design to accommodate interactive technology (music and audio visual) and socialization in dental settings need further research. 

It is crucial to consider that from the very first design of a new dental office or operating room, noise control should be considered, and design should follow such practical issues. For example, we can proceed to install sound insulation between office, scrub-up and sterilization areas and/or the operating room itself. We should also avoid the use of hard sound-reflective ceilings and walls [1], by using sound-absorbing ceilings instead [38]. Further, the use of carpeting and acoustically absorbent ceiling tiles or plants in hallways helps to reduce sound propagation [14]. Flooring used by healthcare units to limit the spread of sound has been also studied [82]. When comparing three types of flooring, it was concluded that a significant difference was found for sound levels between flooring type for equivalent continuous sound levels. Carpet tile performed better for sound attenuation by absorption, reducing sound levels by 3.14 dBA. Carpet tile provides sound absorption that affects sound levels and influences occupant’s perceptions of environmental factors that contribute to the quality of the indoor environment but is suggested only for waiting areas in dental settings. Communities are increasingly imposing bylaws, including the limitation of floor impact sound, minimum thickness of floors and floor soundproofing solutions in the construction of buildings or later in the renovation before establishing the dental units [83]. Further, openings should be covered with soundproof windows, frames, and doors with at least double glass to control the external noises sufficiently. Overall, when designing dental structures, it is obligatory to consider all structural parameters and design characteristics as well as types of acoustic insulating materials such as acoustic putty, sealants, caulk, plaster, wallpapers, sprays or paint and concrete types [84].

Other measures to reduce noise sources include placing compressors away from the dental operatory or within a metal cabinet (such as the M series, Jun Air International Corporation, Japan). The latter has been advocated to reduce noise by 75% to 47–60 dBA [33]. We can also use an anti-vibration soundproof floor mat underneath the compressor to reduce the transmission of noise and vibrations [85]). Additionally, closing the doors when operating is a simple and effective measure for noise reduction within a healthcare unit [86]. Other measures that might work at this direction are also reported, such as verbal and visual alarm reminders for staff, posting quiet signs for patients, and limiting electrical equipment through the waiting areas or the operational office [86].

Architectural design of modern dental offices should consider the use of “service corridors” driving to operational rooms with specific sound isolation walls or sound absorber panels in the walls or ceilings that can distinguish the waiting area from the unit [87] or the different floors of the building. In this way, a stress-free atmosphere in the waiting area with soft music from hidden sound sources can remain undisturbed by the work in the operational room. Finally, within the operational room, equipment that is not used should be kept in cupboards made of soundproof materials mentioned above or of new sustainable ones to lower the environmental impact. There is, for example, an increasing interest for novel ideas to recycle wasted materials such as discarded facemasks for thermal insulation and sound absorption for buildings, replacing the synthetic or petrochemical insulation materials [88]. In addition, in the field of fabrics for furniture or curtains, soundproof rock or stone wool insulation fabrics are suggested [85,89].

Finally, proper maintenance of the handpieces is also important to function well under demanding cutting procedures and to reduce performance noise even after 30 months [90]. Noise appears to be a useful indicator of imminent bearing failure of these cutting instruments. Thus, assiduous adherence to manufacturers’ directions for cleaning and lubrication should contribute to increased bearing life and less noise propagation.

Finally, the method that we can apply to differentiate the perception of the person towards sound and especially noise so that it creates less disturbances is termed “white noise”. White noise is a signal whose spectrum has equal power within any equal interval of frequencies [91], and it is produced by combining sounds of all different frequencies together. In addition, when several distinct auditory signals are presented simultaneously, it is often difficult for the human ear to distinguish or discriminate between them. This phenomenon, known as masking, accounts for the difficulty experienced in hearing others talk in the presence of loud background noise [1]. To minimize noise disturbances, introducing white noise through a sound-masking system—consisting of a central electronic controller and several emitters (speakers)—can be helpful. This constant, low-level background sound fills in the gaps between louder, intermittent noises, making them less noticeable [14]. An easy way to take advantage of the positives of masking is to use background calming music [18] that patients could listen to over headphones to block background noises [37].

## 8. Discussion

The literature reveals that most dental professionals do not take any actions against noise at work. Particularly, most of them do not complain or change, either noisy equipment or the workplace. This is probably because they are familiar with the acoustical environment in dental hospitals, due to their studies in dental schools. It is likely that they do not consider the frequency or the duration of high-level sounds in their everyday working place. Such a noisy workplace would significantly increase the dissatisfaction of dental professionals if they were to know it before opening their own dental office. However, some dental professionals take physical protective measures, such as earplugs [19]. Dentists can use the same solution as performing studio musicians such as the custom-filtered musician’s earplug, which allows for accurate hearing but at lower loudness levels, resulting in a smooth, flat attenuation from 9 to 25 dBA [33]. Regarding dentists and dental assistants, it was found that the aspirator, not the rotating instruments, was the most intense source of noise in the office [21]. The noise of the surgical suction can be reduced through innovative modifications and designs [92]. Another suggestion is that suctions should be used as little as possible and turned off completely when not required [1]. After the COVID-19 pandemic, the use of a rubber dam was universally suggested in safety protocols [93,94], thus diminishing the need for constant use of suction during the whole operational procedure.

Overall, reducing noise in a workplace such as the dental office will contribute to quality services. Dental staff will work more efficiently, and productivity will grow. Time management issues will be better controlled, and the personnel will be happier [95].

Under these conditions, demanding dental procedures can have better outcomes, and patients will be more satisfied. Dentists should show their care for sustainable acoustic solutions in their office. This could be an auto-marketing campaign for a modern dental office committed to sustainability. For example, all electrical equipment should have the relevant acoustic labeling while certificates of soundproof materials used either in furniture or building construction could be advertised as well. New audiovisual equipment with prerecorded music files can be in use while patients can participate in the “noise-free” office by giving their opinions and suggestions in relevant questionnaires found in the waiting areas. 

We need to consider that at least 25% of the European Union population (as estimated) experiences a reduced quality of life due to environmental noise-induced annoyance and that between 5% and 15% of the population suffers serious noise-induced sleep disturbance. This fact is further estimated suggesting that in the EU, environmental noise costs between EUR 13 and 38 billion per annum due to medical costs, lost workdays, reduction in house prices and reduced land use potential. The EU relevant directive [96] (which is also reflected in the approach to noise strategy embodied in the 6th EAP) aims to provide for a common approach to the avoidance, prevention and reduction of the harmful effects of exposure to environmental noise by implementing: (a) strategic noise mapping: determining noise exposure using common noise indicators and methods of assessment; (b) informing the public: providing information on environmental noise and its effects; (c) adopting action plans: based on the results of noise mapping, seeking to reduce noise where necessary and protect environmental noise quality where it is good. The relevant legislation should be encountered worldwide for “noise-free” working places and sustainable modern dental settings.

As dentists, our concern and demand from the construction and dental industry should be the manufacturing of better soundproof “green materials” that can further enhance the initiative of “green dental settings” and the prospect of “noise-controlled” dental working places. We also need to dive deeper into the psychology of the dental patient through the studying of sound while performing dental procedures. In this way, we will be able to help them managing better their emotions, especially negative ones, and make their dental visits more pleasant. Therefore, new studies must continue working in this direction.

## 9. Limitations of the Study

This study was a narrative and non-systematic review, thus possible relevant articles may have not been used. But the overall search was extensive and focused on the main issues addressed about the sound effect in dental offices under the new vision of sustainability for humans in healthcare settings. Novel research on this topic would include the estimation of sound in an academic dental environment with modern devices that guarantee accurate measurements. In addition, estimation of professionals’ and patients’ opinions about the sound levels and the quality of music involved for stress release and relaxation in the dental office are important points for future research.

## 10. Conclusions

There is controversial evidence as to whether music really helps to manage negative emotions and whether it can be applied as therapy or whether it acts as a placebo and is just a pleasant company in the dental office. Positive outcome of most studies, however, suggests that sound control in the dental office will enhance communication, feeling of safety and relaxation to all age groups. Classical or relaxation music will offer distraction to fearful patients during fear-inducing dental procedures. Conducting more research will shed light on the specific spatial and audio–visual design of the waiting areas. This way, we will be able to offer concrete conclusions as to the type of music preferred by patients and staff as well as how they react to diminished noise level while waiting for or accepting therapy services. Finally, modern dental units could be designed using the principles and soundproof materials that already exist or newly designed ones.

## Figures and Tables

**Table 1:** Selected literature on sound effects in dental settings.

Study	Study Type	Methods	Results	Conclusions
Marwah et al., 2005 [68]	Randomized Controlled Clinical Trial	40 children (4–8 years) with no previous dental experience divided in three groups. Group A: control group, Group B: instrumental music group, Group C: nursery rhymes music group	A significant difference (*p* < 0.05) was observed regarding anxiety in groups B and C; higher anxiety levels in group C. A statistically significant (*p* < 0.05) difference was seen between the pulse rates in groups B and C, the anxiety being more in C. The values of oxygen saturation showed minimal variations during all the visits for all the groups, and the results were not statistically significant.	Audio distraction technique decreased the anxiety level but not to a very significant level. Instrumental music was the music of choice. Despite lack of any relief from pain, patients had a positive response to music and wanted to listen to it at their subsequent visits.
Nilsson et al., 2008 [13]	Systematic review	A systematic review of 42 randomized controlled trials of the effects of music interventions in perioperative settings.	Music intervention had positive effects on reducing patients’ anxiety and pain in approximately half of the reviewed studies.	Further research into music therapy and the potential ability of music to reduce peri-operative patient distress is needed.
Ullmann et al., 2008 [51]	Single descriptive study	171 participants answered a questionnaire	Music makes 78.9% of the participants calmer and more efficient. Classical music is the most requested (58%).	Music has a positive effect on the staff working in the operating rooms.
Thoma et al., 2014 [57]	Randomized controlled clinical trial	92 consecutive volunteer patients, N1 = 46, listening to music for 10 min and a control group *n* = 46, waiting in silence	State anxiety levels in the music group decreased significantly after intervention as compared to the control group (1/90) = 8.06; *p* = 0.006).	Listening to music prior to dental hygiene treatment decreases anxiety levels to a greater extent than waiting in silence.
Kühlmann et al., 2018 [69]	Meta-analysis	Systematic literature search- 92 RCTs	Music intervention significantly decreased anxiety and pain compared with controls, equivalent to a decrease of 21 mm for anxiety and 10 mm for pain on a 100 mm visual analogue scale.	Music intervention significantly reduces anxiety and pain in adult surgical patients.
Packyanathan et al., 2019 [6]	Randomized Controlled Clinical Trial	50 patients in Saveetha Dental College were randomly selected and allocated to test group and control group. The test group (N = 25) was subjected to music during extractions and control (N = 25) was not exposed. Dental anxiety levels and hemodynamic changes were assessed before and after extraction.	The control population had elevated hemodynamic changes, as the diastolic pressure rise was significant. In the test population, there was a statistically significant fall in the hemodynamic changes.	Music seems to be a psychological and spiritual way to calm oneself down. Hence, music therapy can be used as an anxiolytic agent for stressful dental procedures.
Oomens et al., 2019 [52]	Systematic review	Systematic literature search—9 studies (212 participants)	Beneficial effects of music were reported on time to task completion, instrument handling, quality of surgical task performance and general surgical performance.	Insufficient evidence to definitively conclude that music has a beneficial effect on surgical performance in the simulated setting
Gupta et al., 2020 [8]	Pilot study	50 adult patients attending the MOS Clinic at Birmingham Dental Hospital. Instrumental music was played for the patient via earphones during MOS treatment. Both physiological and psychological measures of anxiety were recorded using heart rate measurements, patient completed questionnaires and a subjective ten-point anxiety score.	Τhe majority of patients reported music reduced their anxiety levels, pain and discomfort (92%). Almost half of the respondents (48%) reported that music made communication with the dental team easier, and 90% of patients reported that they would request to have music playing during their next dental visit.	Music can be helpful in making patients feel more at ease during dental treatment.
Fu VX et al., 2021 [35]	Systematic review	Systematic literature search—22 prospective studies (3507 participants)	Over half of the surveyed staff found noise levels to be a disturbing stressor and impacted performance negatively.	Although music increased decibel levels in the operation room, attitude of surgical team members toward music during surgery is generally regarded favorable.
